# GPR75: Advances, Challenges in Deorphanization, and Potential as a Novel Drug Target for Disease Treatment

**DOI:** 10.3390/ijms26094084

**Published:** 2025-04-25

**Authors:** Jingyi Han, Jiaojiao Li, Sirui Yao, Zao Wei, Hui Jiang, Tao Xu, Junwei Zeng, Lin Xu, Yong Han

**Affiliations:** 1 Department of Physiology, Zunyi Medical University, Zunyi 563006, China; hjy6619@zmu.edu.cn (J.H.); ljj7147@zmu.edu.cn (J.L.); ysr4047@zmu.edu.cn (S.Y.); wz9971@zmu.edu.cn (Z.W.); jianghui@zmu.edu.cn (H.J.); taoxu@zmu.edu.cn (T.X.); junweizeng@zmu.edu.cn (J.Z.); 2 Department of Immunology, Zunyi Medical University, Zunyi 563006, China

**Keywords:** chemokine ligand 5, deorphanization, G protein-coupled receptor 75, signal transduction, 20-hydroxyeicosatetraenoic acid

## Abstract

G protein-coupled receptor 75 (GPR75), a novel member of the rhodopsin-like G protein-coupled receptor (GPCR) family, has been identified across various tissues and organs, where it contributes to biological regulation and disease progression. Recent studies suggest potential interactions between GPR75 and ligands such as 20-hydroxyeicosatetraenoic acid (20-HETE) and C-C motif chemokine ligand 5 (CCL5/RANTES); however, its definitive endogenous ligand remains unidentified, and GPR75 is currently classified as an orphan receptor by International Union of Basic and Clinical Pharmacology (IUPHAR). Research on GPR75 deorphanization has underscored its critical roles in disease models, particularly in metabolic health, glucose regulation, and stability of the nervous and cardiovascular systems. However, the signaling pathways of GPR75 across different pathological conditions require further investigation. Importantly, ongoing studies are targeting GPR75 for drug development, exploring small molecule inhibitors, antibodies, and gene silencing techniques, positioning GPR75 as a promising GPCR target for treating related diseases. This review summarizes the recent advancements in GPR75 deorphanization research, examines its functions across tissues and systems, and highlights its links to metabolic, cardiovascular, and neurological disorders, thereby providing a resource for researchers to better understand the biological functions of this receptor.

## 1. Introduction

G protein-coupled receptors (GPCRs) constitute the largest and most significant family of membrane protein receptors in the human genome. Based on sequence homology and functional similarities, GPCRs are grouped into six classes: Class A (rhodopsin-like receptors), Class B (secretin receptors), Class C (metabotropic glutamate receptors), Class D (fungal mating pheromone receptors), Class E (cyclic adenosine monophosphate receptors), and Class F (frizzled/smoothened receptors) [1]. In the human genome, only Classes A, B, C, and F are present, totaling over 800 types of GPCRs. Among these, Class A (rhodopsin-like receptors) comprises the majority, with 719 types distributed across 19 subfamilies (A1–A19), serving as potential targets for most clinically used drugs [2]. Yang et al. [3] reported that approximately 500 GPCR-targeting drugs are directed at Class A receptors, with 75% targeting amine receptors and 10% targeting peptide-ligand receptors. These drugs are used to treat various conditions, including pain, allergies, cardiovascular and pulmonary diseases, depression, migraines, glaucoma, Parkinson’s disease, schizophrenia, and cancer-related fatigue. According to the latest International Union of Basic and Clinical Pharmacology (IUPHAR) pharmacology guide [4], 197 receptors in the Class A rhodopsin-like receptor family have identified endogenous ligands, excluding receptors related to olfactory, visual, taste, and vomeronasal functions. However, 87 receptors in this family lack identified endogenous ligands and are termed “orphan GPCRs”. For these orphan receptors, researchers continuously pursue “deorphanization” studies. At least one potential endogenous ligand has been reported for 54 orphan receptors, with possible associations with various diseases.

G protein-coupled receptor 75 (GPR75) is listed by IUPHAR as an orphan receptor with relevant research findings [4]. Initial studies suggested that GPR75 might interact with the C-C motif chemokine ligand 5 (CCL5, also known as RANTES), influencing nervous system functions [5]. Recent studies indicate that GPR75 may also bind to 20-hydroxyeicosatetraenoic acid (20-HETE), a metabolite of cytochrome P450 (CYP450) enzymes [6,7], highlighting its roles in the nervous, cardiovascular, and endocrine systems and its potential involvement in neurodegenerative diseases, hypertension, and metabolic syndrome. However, debate continues regarding GPR75’s endogenous ligands. This article reviews recent advances in GPR75 deorphanization research, discusses its roles and possible mechanisms in various diseases, and aims to provide a reference for scholars to further explore the biological functions of this receptor.

## 2. Overview of G Protein-Coupled Receptor 75 (GPR75)

**Figure 1:**
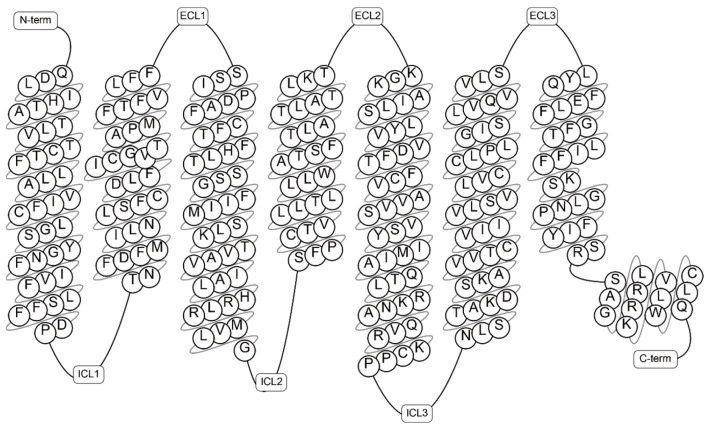
Topology of GPR75. The transmembrane region of GPR75 consists of 7TM, including 3ECL located outside the receptor and involved in binding ligands; 3ICL located inside the receptor and involved in interacting with G proteins or other signaling molecules to transmit signals; and the N-terminal region, which is located outside the membrane, and the C-terminal region, located inside the cell (https://gpcrdb.org/protein/gpr75_human, accessed on 12 February 2025).

In 1999, Tarttelin et al. [8] first identified the *GPR75* gene in the human genome, located on chromosome 2p16. It encodes a 540-amino acid protein with a molecular weight of 59.4 kDa. Protein sequence analysis reveals that GPR75 exhibits typical GPCR characteristics, including a seven-transmembrane (7TM) domain, three extracellular loops (3ECL), three intracellular loops (3ICL), an N-glycosylation site at the N-terminus, and serine/threonine phosphorylation sites at the C-terminus. The topology of GPR75 is shown in Figure 1. (Diagrams available at https://gpcrdb.org/protein/gpr75_human, accessed on 12 February 2025). Comparative analysis shows that this receptor shares transmembrane domain homology with the neuropeptide Y receptor in Caenorhabditis elegans, the rat galanin receptor type 3, and the porcine growth hormone secretagogue receptor type 1b [8,9], offering a foundation for further research on the functions of the *Gpr75* gene and its encoded protein, as well as its conservation and variability across species.

Tarttelin et al. [8] mapped the *GPR75* gene within the Doyne’s honeycomb retinal dystrophy (DHRD) region on chromosome 2p16 and reported that GPR75 transcripts are primarily expressed in the retinal pigment epithelium and various brain regions. However, no mutations in this gene were found in patients with DHRD-related retinal disorders. Sauer et al. [10] conducted a mutation screening of the GPR75 coding sequence in 535 age-related macular degeneration (AMD) patients and 252 control subjects from Germany, the United States, and Italy, but found no significant association between GPR75 sequence variations and AMD susceptibility. Therefore, early studies provided limited insights into this gene’s biological function. In related research, Voogdt et al. [11] examined pattern recognition receptors (PRRs) in avian species, noting that *Gpr75* is located adjacent to the Toll-like receptor TLR15 in the chicken genome. Given PRRs’ roles in immune homeostasis and antimicrobial defense, this finding suggests potential shared biological functions between *Gpr75* and *Tlr15*, although definitive evidence is lacking. More recent studies have classified GPR75 as a Gα_q_ protein-coupled receptor within the Class A rhodopsin-like family [5,6,12], with a broad expression profile across tissues and organs, including the retina, brain, heart, pancreas, lungs, kidneys, uterus, and prostate. Its functions and associations with various diseases are gradually emerging, though its biological roles and disease mechanisms remain to be fully elucidated. At present, the endogenous ligands, agonists, and antagonists for GPR75 have not been conclusively identified, and some controversy persists in this area.

## 3. Deorphanization Research of GPR75

### 3.1. Identification of CCL5 as the First Potential Ligand for GPR75

Identifying the endogenous ligand for GPR75, a novel GPCR, is critical for understanding its biological functions and disease associations. In 2006, Ignatov et al. [5] first reported that the inflammatory chemokine CCL5 might serve as an endogenous ligand for GPR75. CCL5 is a CC subfamily chemokine primarily expressed in T lymphocytes and monocytes [13]. As a ligand, CCL5 binds primarily to members of the CC chemokine receptors (CCR) family, including CCR1, CCR3, and CCR5, with the highest affinity for CCR5 [14]. CCR5, a Class A rhodopsin-like GPCR, has been shown to regulate cell proliferation, migration, and angiogenesis, thereby influencing conditions such as inflammation, tumorigenesis, and viral infections [15]. Ignatov et al. [5] demonstrated that in HEK293 cells overexpressing *GPR75*, as well as in mouse hippocampal neurons (HT22 cells) and African green monkey kidney cells (CV-1 cells) that endogenously express GPR75, CCL5 treatment induced inositol 1,3,5-triphosphate (IP_3_) accumulation, elevated intracellular Ca^2+^ levels, and activated AKT/MAPK signaling pathways. IP_3_ accumulation and Ca^2+^ elevation are hallmarks of Gα_q_ protein activation in GPCR signaling [16]. Additionally, CCL5-induced responses were suppressed by PLC inhibitor U73122, or PI3K inhibitor wortmannin, indicating that CCL5 activates GPR75 in neuronal cells via the Gα_q_ signaling pathway. Dedoni et al. [12] further explored this interaction in rat cortical neurons and neuroblastoma SH-SY5Y cells, finding that CCL5 triggers a signaling cascade involving AKT, ERK1/2, and GSK3β through a Gα_q_-mediated, PTX-insensitive mechanism. Inhibition by the CCR1 inhibitor BX471, CCR3 inhibitor SB328437, or CCR5 inhibitor DAPTA did not block CCL5-induced phosphorylation of AKT, GSK3β, and ERK1/2, but CRISPR-Cas9 knockdown of *Gpr75* effectively abolished these effects, demonstrating GPR75’s role in mediating CCL5’s signaling. The study also observed GPR75 internalization and activation following CCL5 binding. Beyond the nervous system, Liu et al. [17] reported similar findings in pancreatic β-cells. Their work on mouse and human islet β-cells revealed that while known CCL5 receptors (CCR1, CCR3, and CCR5) were minimally expressed, both GPR75 and CCL5 were significantly expressed. Knockdown of *Gpr75* via siRNA effectively blocked CCL5-induced Ca^2+^ increases and insulin secretion. Similarly, Gençoğlu et al. [18] assessed the effects of GPR75, GPR54, GPR56, and cannabinoid receptors CB1R and CB2R on pancreatic β-cell function, finding that CCL5–GPR75 interaction stimulated insulin secretion and improved blood glucose homeostasis in MIN6 β-cells. Recently, D’Addario et al. [19] studied a hypoxia-induced mouse model of pulmonary hypertension (PH), observing that chronic hypoxia upregulated GPR75 expression in the alveoli, airways, and pulmonary arteries, along with increased CCL5 levels. Both global *Gpr75* knockout (*Gpr75*^−^/^−^) and endothelial cell-specific knockout (EC-*Gpr75*^−^/^−^) completely blocked hypoxia-induced contraction in intrapulmonary arteries (IPAs) and prevented PH development. Interestingly, while *Gpr75* knockout did not significantly affect hypoxia-induced IP_3_ production (a marker for Gα_q_ activation), it significantly increased cAMP levels (a marker for Gα_i_ activation). Additionally, *Gpr75* knockout inhibited the CCL5-induced decrease in cAMP levels, suggesting that CCL5 promotes PH via GPR75’s Gα_i_ signaling pathway, contrasting previous findings where CCL5 activated GPR75 through Gα_q_ signaling. This difference may be attributed to GPCR biased signaling or complex regulatory mechanisms.

Biased signaling refers to the phenomenon in which ligands or receptors selectively activate specific signaling pathways (e.g., G protein- or β-arrestin-mediated pathways) rather than engaging all downstream effectors simultaneously. Lefkowitz et al. [20] revealed the role of β-arrestin in GPCR signaling and its segregation from G protein signaling, laying the conceptual foundation for biased signaling research. Intracellular signaling operates through two distinct dimensions mediated by G proteins and β-arrestin, with ligands or receptors exhibiting preferential activation of one pathway while minimally activating or even inhibiting the other [21]. The central significance of biased signaling lies in its transformation of the traditional binary “activation/inhibition” paradigm into a multidimensional framework of “pathway-selective regulation”, thereby offering broad opportunities for therapeutic efficacy optimization, side effect reduction, and mechanistic exploration [22]. Recent studies suggest that this bias is not limited to the G protein versus β-arrestin pathways. Certain receptors, when activated by specific ligands, exhibit bias toward different G protein subtypes (e.g., Gα_i_, Gα_s_, Gα_q_) [23], a phenomenon that may arise from variations in receptor structural features or ligand–receptor interaction modalities. For instance, the endothelin type B receptor (ETB) has been shown to differentially activate Gα_s_, Gα_i_, and Gα_q_ subtypes, accompanied by specific conformational changes during the activation of distinct G proteins [24]. It remains unclear whether GPR75 exhibits similar biased signaling characteristics, which warrants further investigation.

Currently, five studies support the hypothesis that CCL5 may act as an endogenous ligand for GPR75, though none have directly observed binding between CCL5 and GPR75. Southern et al. [25] conducted a ligand screening experiment using β-arrestin recruitment assays for various orphan receptors, including GPR75, testing 10,500 candidate ligands. Although several ligand–receptor pairs were confirmed (e.g., GPR35 with kynurenic acid, GPR84 with capric acid, and MRGPRX2 with cortistatin-14), no interaction was observed between GPR75 and CCL5. In summary, whether CCL5 exerts its effects by activating specific G protein subtypes remains controversial. Additionally, β-arrestin recruitment experiments have not confirmed whether its downstream signaling is mediated by β-arrestin-biased signaling. Thus, the issue of GPR75’s endogenous ligand remains unresolved.

### 3.2. 20-HETE Is a High-Affinity Potential Ligand for GPR75

20-HETE is a vasoactive substance produced from arachidonic acid (AA) metabolism, catalyzed by CYP4A/4F enzymes within the CYP450 family [7,26]. Recent clinical and animal studies have implicated 20-HETE in cardiovascular diseases, including hypertension, stroke, ischemic cardiomyopathy, and heart failure [27,28,29,30,31]. As a potent vasoconstrictor, 20-HETE exhibits effects similar to angiotensin II (Ang II) and endothelin-1 (ET-1), playing a key role in regulating vascular tone, blood pressure, and vascular remodeling in both physiological and pathological contexts [32]. While specific receptors mediate the actions of substances like Ang II, the receptor and underlying mechanism for 20-HETE remain less clear. Early studies observed that structural analogs of 20-HETE, such as 20-hydroxyeicosa-6(Z),15(Z)-dienoic acid (20-HEDE) and 20-hydroxyeicosa-6(Z),15(Z)-dienoylglycine (20-HEDGE), could counteract its vasoconstrictive effects, suggesting the involvement of specific receptor-mediated mechanisms [32,33]. Additionally, 20-HETE’s vasoconstrictive and diuretic effects have been linked to the PLC/PKC pathway, and its effects on vascular smooth muscle cell (VSMC) migration, endothelial dysfunction, and inflammation appear to involve the c-Src/EGFR pathway [34], which is commonly activated by GPCRs [35]. These findings strongly indicate the presence of a 20-HETE receptor. However, due to its high lipophilicity and rapid esterification into phospholipids, studying whether 20-HETE binds to membrane receptors remains challenging.

In 2017, Garcia et al. [6] reported GPR75 expression in the vascular system and identified 20-HETE as a ligand that binds to and activates this receptor. Their findings show that 20-HETE induces endothelial dysfunction and VSMC contraction through GPR75. In VSMCs, 20-HETE activation of GPR75 led to Gα_q_ protein dissociation and subsequent activation of the PLC pathway, triggering IP_3_ production. This IP_3_ generation, a hallmark of GPCR activation, stimulates PKC pathways and IP_3_ receptor channels, raising intracellular Ca^2+^ levels and promoting VSMC contraction. Similarly, in endothelial cells, 20-HETE’s interaction with GPR75 activated G protein-coupled receptor kinase interactor 1 (GIT1), inducing EGFR phosphorylation and activating the NF-κB pathway, which increased angiotensin-converting enzyme (ACE) expression and contributed to endothelial dysfunction. Notably, in their study, CCL5 treatment failed to trigger the dissociation of the Gα_q_ protein from GPR75, indicating that CCL5 does not mediate intracellular signaling through G protein activation. Furthermore, Southern et al. [25] also observed no recruitment of β-arrestin by CCL5, suggesting that it does not act via biased activation of the β-arrestin signaling pathway. These findings challenge the hypothesis that CCL5 functions as a typical agonist for GPR75 in certain cellular contexts.

The discovery of the 20-HETE–GPR75 axis in the vascular system provides critical insights into GPR75’s biological functions and elucidates receptor-mediated mechanisms of 20-HETE’s actions. Recent studies, including those from our research group [36], have confirmed the 20-HETE/GPR75 axis’s significant role across various tissues and disease contexts. Cárdenas et al. [37] reported GPR75 expression in prostate cancer PC-3 cells, where 20-HETE significantly increased hydrogen peroxide-inducible clone-5 (HIC-5) protein expression, a key downstream GPCR signaling molecule, as well as EGFR, NF-κB, AKT, and p38 MAPK phosphorylation. Silencing *Gpr75* or using the water-soluble 20-HETE antagonist N-disodium succinate-20-hydroxyeicosa 6(Z),15(Z)-diencarboxamide (AAA) reversed these effects. Similarly, Gilani et al. [38] observed in differentiated 3T3-L1 adipocytes that AAA blocked the inhibitory effect of 20-HETE on insulin receptor Tyr972 phosphorylation, thereby ameliorating the resulting impairment of insulin signaling. The proposed mechanism is that 20-HETE binds to GPR75, thereby activating the IP_3_/DAG signaling cascade, increasing intracellular Ca^2+^ concentration, and initiating the PKC pathway, which plays a key role in insulin synthesis and secretion. Recently, Chen et al. [39] found in human fetal lung fibroblast cell line MRC5 and mouse fibroblast cell line L929 that 20-HETE upregulates GPR75 expression, and that knocking down *Gpr75* effectively inhibits the 20-HETE-induced expression of tumor immune evasion factors, such as α-smooth muscle actin (α-SMA), fibroblast activation protein-α (FAP), programmed death-ligand 1 (PD-L1), interleukin 6 (IL-6), and transforming growth factor-β (TGF-β). Pascale et al. [40] characterized the pharmacological properties of 20-HETE and its agonists targeting GPR75 in the EA.hy926 endothelial cell line by measuring intracellular Ca^2+^ changes, revealing that 20-HETE and agonists like 5,14-HEDE and 5Z-HOTE can activate GPR75, whereas 20-HETE antagonists, such as 20-SOLA and AAA, exhibit significant inhibitory effects on GPR75 activity.

Pascale et al. [41] confirmed through surface plasmon resonance that 20-HETE is a high-affinity ligand for GPR75. Binding of 20-HETE to GPR75 induces an increase in intracellular free Ca^2+^ concentration and IP-1 accumulation in HTLA cells, an effect that can be blocked by the 20-HETE antagonist AAA. Notably, the study also revealed that although CCL5 increases Ca^2+^ concentration in HTLA and human endothelial cells, this effect is independent of GPR75. In fact, overexpression of *Gpr75* reduces CCL5-induced IP-1 accumulation and Ca^2+^ elevation, while CCL5 treatment significantly inhibits the 20-HETE-triggered increases in these signals. Moreover, β-arrestin recruitment assays via the PRESTO-Tango method showed that CCL5 blocks 20-HETE-induced β-arrestin recruitment, suggesting that 20-HETE is a high-affinity ligand for GPR75, whereas CCL5, acting as a low-affinity ligand, competitively hinders 20-HETE’s activation of GPR75. However, contrary to Pascale et al. [41], Jiang et al. [42] reported that in HEK293T cells overexpressing *GPR75*, neither 20-HETE nor CCL5 were detected in β-arrestin recruitment assays using the PRESTO-Tango method. Instead, the structural analog of 20-HETE, 5,14-HEDE, has demonstrated appropriate activation of GPR75. This may be attributed to the susceptibility of 20-HETE to oxidation, thereby diminishing its functional activity. Both 5,14-HEDE and 20-HETE are long-chain hydroxylated unsaturated fatty acids with similar structures, allowing them to retain essential features for binding and activating GPR75, thus demonstrating activation at high experimental concentrations. However, further investigation is needed to confirm these mechanisms.

In the cardiovascular system, Agostinucci et al. [43] employed a transgenic mouse model with vascular smooth-muscle-targeted overexpression of *Cyp4a12* to induce hypertension and observed that in vivo administration of the 20-HETE antagonist AAA alleviated 20-HETE-induced Rho kinase activity by blocking myosin light chain phosphorylation and reducing VSMC sensitivity to Ca^2+^. Tunctan et al. [44] applied a 20-HETE analog, 5,14-HEDGE, in a rat model of lipopolysaccharide (LPS)-induced hypotension and tachycardia and observed that it activates GPR75. Treatment with 5,14-HEDGE in rat arteries activated GPR75 downstream pathways, including Gα_q/11_/PKCα/MaxiKβ, GIT1/PKCα/MaxiKβ, GIT1/c-Src/MaxiKβ, and GIT1/c-Src/EGFR, while AAA effectively reversed these effects. In the heart, our research demonstrated that GPR75 is expressed in neonatal rat cardiomyocytes and H9c2 cells and that *Gpr75* knockdown significantly reduced the 20-HETE-induced increases in IP_3_ content and Ca^2+^ levels, as well as its pro-apoptotic effects on cardiomyocytes [45,46]. Recently, we found that the 20-HETE/GPR75 axis is involved in Ang II-induced cardiomyocyte hypertrophy. Inhibition of 20-HETE synthesis with HET0016 or blockade of GPR75 with AAA markedly suppressed both the upregulation of hypertrophic genes ANP and BNP and the increase in cell size induced by Ang II [36].

In the nervous system, Gonzalez-Fernandez et al. [47] recently investigated the expression of CYP4A and GPR75 in brain sections from Dahl salt-sensitive and *Cyp4a1* transgenic rats using immunofluorescence. They observed that both CYP4A and GPR75 are expressed in multiple areas of the brain, including the neocortex, entorhinal cortex, hippocampus, thalamus, and hypothalamus, as well as in cerebral vasculature, VSMCs, and perivascular cells. This suggests that the 20-HETE/GPR75 axis may play an important role in cerebrovascular diseases and cognitive functions.

**Figure 2:**
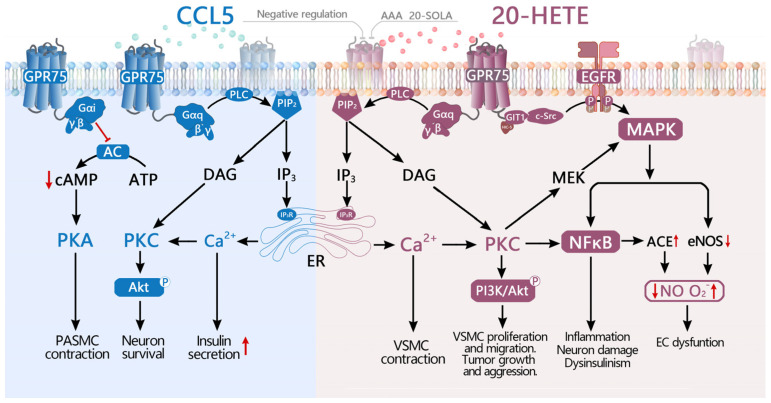
Overview of CCL5 and 20-HETE as GPR75 ligands and signaling pathways. CCL5, the first identified ligand for GPR75, enhances insulin secretion by activating the Gα_q_/PLC pathway, which elevates IP_3_ and Ca^2+^ levels. The CCL5/GPR75 axis also stimulates the DAG/PKC pathway, leading to the activation of AKT and MAPK signaling, which inhibits Aβ-induced neuronal apoptosis and confers neuroprotection. Additionally, in pulmonary artery smooth muscle cells (PASMCs), CCL5 decreases cAMP levels via Gα_i_ signaling, thereby inducing contraction. It also functions as a negative regulator of 20-HETE/GPR75 interactions. In contrast, 20-HETE, a high-affinity ligand for GPR75, induces contraction in vascular smooth muscle cells (VSMCs) by activating the Gα_q_/PLC pathway, leading to increased IP_3_ and Ca^2+^ levels. Moreover, the 20-HETE/GPR75 axis activates the PI3K/AKT pathway to promote cell proliferation, migration, and tumor invasiveness. It also induces EGFR phosphorylation via GIT1, which activates MAPK and NF-κB signaling and contributes to neuronal damage and impaired insulin secretion. In addition, the 20-HETE/GPR75 axis triggers c-Src/EGFR/MAPK signaling, resulting in increased ACE expression, eNOS uncoupling, and consequent endothelial dysfunction. ↑: Upregulation. ↓: Downregulation. Abbreviations: CCL5: C-C motif chemokine ligand 5; 20-HETE: 20-hydroxyeicosatetraenoic acid; EGFR: epidermal growth factor receptor; ER: endoplasmic reticulum; AC: adenylil cyclase; GIT1: G protein-coupled receptor kinase interactor 1; PIP_2_: phosphatidylinositol 4,5-bisphosphate; HIC-5: hydrogen peroxide-inducible clone-5; c-Src: cellular Src; IP_3_: inositol 1,3,5-triphosphate; VSMC: vascular smooth muscle cell; EC: endothelial cell; PASMC: pulmonary artery smooth muscle cell.

In summary, early studies by Ignatov, Liu, and Dedoni et al. [5,12,17] proposed GPR75 as a receptor for CCL5 based on CCL5-induced IP_3_ accumulation and Ca^2+^ increase. However, these studies did not directly assess CCL5 binding to GPR75 and were not confirmed in subsequent β-arrestin recruitment assays. Conversely, Garcia, Cárdenas, and Gilani et al. [6,37,38] identified 20-HETE as a potential ligand for GPR75 across various tissues, and Pascale et al. [41] proposed that CCL5 may negatively regulate 20-HETE’s interaction with GPR75. Figure 2 illustrates the current research findings and signaling pathways associated with CCL5 and 20-HETE as potential GPR75 ligands.

Although the endogenous ligand of GPR75 remains uncertain, ongoing studies of its biological functions and disease associations suggest that GPR75 could emerge as an important GPCR drug target for related diseases.

## 4. Function of GPR75 and Involvement in Diseases

### 4.1. The Role of GPR75 in the Nervous System

CCL5, as a potential GPR75 ligand, has been shown to significantly influence nervous system function. Previous studies demonstrated that CCL5, by binding to the CCR5 receptor and activating associated pathways, impacts learning and memory, brain development, neuroinflammation, and neurodegenerative diseases, such as Alzheimer’s disease (AD) [48]. However, the specific role of the CCL5/CCR5 axis in AD remains unclear, with contradictory findings. Some studies have reported elevated CCL5 and CCR5 levels in AD patients, correlating with amyloid β-protein (Aβ) deposition and increased secretion of pro-inflammatory factors IL-6 and TNF-α [49]. CCR5 inhibition or antagonism has been shown to reduce neuroinflammation, potentially alleviating AD pathology [50]. Conversely, other studies have observed decreased CCR5 expression and lower CCL5 levels in AD animal models and in the peripheral blood of AD patients [51,52]. In vitro experiments have suggested a neuroprotective effect of CCL5, enhancing neuronal survival and increasing cellular tolerance to sodium nitroprusside and thrombin toxicity [53]. Aβ accumulation in neural tissue is neurotoxic and closely linked to neuronal degeneration in AD patients [54]. Ignatov et al. [5] demonstrated that in hippocampal HT22 neurons expressing GPR75, CCL5 effectively inhibited Aβ-induced apoptosis and activated the AKT and MAPK signaling pathways, effects further enhanced by *Gpr75* overexpression. Dedoni et al. [12] found in SH-SY5Y neuroblastoma cells that CCL5 activates the AKT and MAPK pathways via GPR75 through the Gα_q_ protein pathway, thereby exerting anti-apoptotic and neuroprotective effects.

In contrast, 20-HETE, another potential GPR75 ligand, has been shown to be neurotoxic and is associated with stroke, cognitive impairment, and AD development [55]. Enhanced neuronal activity requires increased local blood flow, a process known as neurovascular coupling (NVC) [56]. Elevated 20-HETE levels in cerebrospinal fluid and plasma in stroke patients have been linked to post-stroke cerebral blood flow reductions insufficient to meet the demands of increased neuronal activity, thereby impairing NVC. This disruption may underlie 20-HETE’s involvement in neurodegenerative diseases, such as AD, while inhibition of 20-HETE synthesis has been found to alleviate post-ischemic cerebrovascular spasms and reduce infarct size [57,58]. Recently, Gonzalez-Fernandez et al. [47] observed co-expression of *Cyp4a* and *Gpr75* in several regions of the brain, including the neocortex and entorhinal cortex, as well as in endothelial cells, VSMCs, and pericytes in cerebral vessels, suggesting that 20-HETE may influence VSMC and pericyte function via a GPR75-mediated paracrine mechanism. Ma et al. [59] recently revealed that 20-HETE activates microglia by binding to GPR75, contributing to the inflammatory response following traumatic brain injury (TBI) in immature brains. Silencing the *Gpr75* gene or applying HET0016 effectively suppressed the activation of the c-Src/EGFR/NF-κB pathway and reversed the 20-HETE-induced inflammatory response in BV-2 microglial cells. These findings suggest that the 20-HETE/GPR75 axis plays a critical role in inflammation following brain injury.

Epoxyeicosatrienoic acids (EETs), another class of bioactive lipids derived from arachidonic acid via the CYP450 epoxygenase pathway (CYP2C/2J), degrade into inactive dihydroxyeicosatrienoic acids (DHETs) via soluble epoxide hydrolase (sEH) [7,60]. EETs, unlike 20-HETE, exhibit vasodilatory, anti-inflammatory, and antioxidant effects [61]. Recently, Horat et al. [62] reported that GPR75 plays a crucial role in mediating the neuroprotective effects of EETs. Using TPPU, a selective sEH inhibitor that maintains EET levels, they observed significant symptom improvement in a mouse model of chronic autoimmune encephalomyelitis induced by MOG35-55 peptide/PT. These beneficial effects were closely linked to inhibition of GPR75-mediated Gα_q_/AP-1 signaling, which reduced pro-inflammatory activity, suggesting that GPR75 and its downstream pathways play a crucial role in demyelinating nervous system diseases. Speidell et al. [63] investigated the role of GPR75 in emotional and cognitive functions using a *Gpr75* knockout mouse model. They found that knockout mice exhibited increased anxiety-like behavior and impaired recall in trace fear conditioning tasks, potentially related to altered presynaptic structural protein regulation in the hippocampus due to GPR75 absence, affecting neuronal transmission function. These results provide behavioral evidence supporting GPR75’s role in brain function.

Although the *GPR75* gene and its transcript were initially identified in the retinal pigment epithelium, studies by Sauer et al. [10] found no direct association between this gene and retinal diseases or other ocular conditions. Additional research has linked the 2p16 region, where *GPR75* is located, to autosomal dominant juvenile primary open-angle glaucoma (POAG) in two large Chinese families [64]. However, sequencing analysis of *GPR75* did not reveal disease-associated mutations in these families, suggesting GPR75 may not be a direct causative factor for POAG in these cases. Recently, Vasudevan et al. [65] reported that in *Gpr75* knockout mice, the absence of GPR75 led to progressive cone photoreceptor cell loss and dysfunction with age, indicating GPR75’s crucial neuroprotective role in maintaining retinal cone photoreceptor function.

### 4.2. The Impact of GPR75 on Cardiovascular System Function

In the vascular system, GPR75 is expressed in VSMCs and endothelial cells and is implicated in hypertension development in various animal models. In a *Cyp4a12* transgenic mouse model of 20-HETE-dependent hypertension, *Gpr75* knockdown significantly reduced doxycycline-induced blood pressure elevation, decreased ACE expression, and improved endothelial dysfunction [6]. In an Ang II-dependent malignant hypertension model using *Cyp1a1-Ren-2* transgenic rats, blocking GPR75 with AAA effectively reversed I3C-induced malignant hypertension, which is associated with reduced Ang II levels in plasma and kidneys [66]. Additionally, Agostinucci et al. [43] developed a VSMC-targeted *Cyp4a12* transgenic mouse model exhibiting enhanced VSMC contractility and 20-HETE-dependent hypertension; AAA treatment effectively reduced blood pressure and improved acetylcholine-induced vasodilation. In a rat septic shock model, Tunctan et al. [44] showed that the 20-HETE analog 5,14-HEDGE activated GPR75, suppressing LPS-induced vascular hyporeactivity, hypotension, tachycardia, and arterial inflammation, effects that were reversible with AAA. Studies in spontaneous hypertensive rat models have further revealed that combining AAA with the 14,15-EET analog EET-A exhibits antihypertensive effects, enhances nitric oxide metabolite excretion, and improves endothelial function [67,68]. Recently, D’Addario et al. demonstrated that global or endothelial-specific *Gpr75* knockout alleviated hypoxia-induced PH, underscoring GPR75’s role in hypoxia-induced PH pathogenesis. In the heart, GPR75’s role remains underexplored. Our preliminary studies [45,46] showed GPR75 expression in neonatal cardiomyocytes and H9c2 cells, where *Gpr75* knockdown significantly attenuated 20-HETE-induced cardiomyocyte apoptosis. Additionally, our recent work revealed that the 20-HETE/GPR75 axis plays a critical role in Ang II-induced cardiac hypertrophy [36]. These findings offer new insights into GPR75’s cardiac function, though further investigation is required to fully elucidate the underlying molecular mechanisms. Collectively, these studies strongly suggest GPR75’s involvement in cardiovascular diseases, particularly in blood pressure regulation and endothelial function. Knocking down *Gpr75* expression or blocking its activity may present a promising therapeutic target for hypertension and related cardiovascular conditions.

### 4.3. The Role of GPR75 in Metabolic Disorders

Two potential GPR75 ligands, CCL5 and 20-HETE, play significant roles in metabolic syndrome. Studies have shown that CCL5 inhibits glucose-stimulated GLP-1 and GLP-2 secretion, leading to impaired pancreatic islet function in mice [69]. Elevated plasma CCL5 levels have been observed in overweight and obese individuals, correlating positively with body mass index (BMI) and body fat percentage. The activation of the CCL5/CCR5 axis has been linked to exacerbated obesity and insulin resistance [70,71]. Liu et al. [17] reported that CCL5, acting as a GPR75 ligand, stimulates insulin secretion and improves glucose homeostasis in lean and insulin-resistant mice. Gençoğlu et al. [18] further confirmed GPR75’s role in enhancing insulin secretion in MIN6 pancreatic β-cells. Similarly, 20-HETE levels correlate positively with BMI, hyperglycemia, and plasma insulin levels, thereby contributing to weight gain, elevated fasting glucose, and insulin resistance [72]. Gilani et al. [38] demonstrated that GPR75 activation is essential for 20-HETE’s role in obesity-related diabetes and insulin secretion pathway disruptions. These findings underscore GPR75’s potential as a novel therapeutic target for metabolic syndrome and associated conditions, including obesity and type 2 diabetes. In addition, recent research by Hardwick et al. [73] revealed that the 20-HETE/GPR75 axis plays a significant role in the development and progression of metabolic-dysfunction-associated steatosis liver disease (MASLD). From the stage of fatty liver to cirrhosis, the levels of 20-HETE continue to rise. In contrast, GPR75 mRNA and protein expression increase during fatty liver and steatohepatitis stages but decline significantly in cirrhosis. In the cirrhotic stage, the levels and expression of 20-HETE and GPR75 exhibit an inverse regulatory pattern compared to the early stages of MASLD. The authors suggest that elevated CCL5 levels in cirrhosis may competitively inhibit GPR75 activation by 20-HETE, leading to reduced GPR75 expression, as previously proposed by Pascale et al. [41].

Akbari et al. [74,75] conducted a large-scale exome sequencing study with 645,000 human samples and identified 16 gene mutations, including GPR75, that are highly associated with BMI. Protein truncating variants (PTVs) in GPR75 were detected in approximately 4 per 10,000 individuals, who exhibited an average BMI reduction of 1.8 kg/m^2^, a weight decrease of 5.3 kg, and a 54% lower obesity risk compared to non-carriers [74]. To confirm GPR75’s role in obesity suppression, the study observed that in a high-fat-diet (HFD)-induced obesity model, untreated mice nearly doubled their weight over 14 weeks, whereas heterozygous (*Gpr75*^+/−^) mice gained 25% less and homozygous (*Gpr75*^−/−^) mice gained 45% less weight. Furthermore, mice with one or both copies of *Gpr75* deleted showed improved glucose tolerance and insulin sensitivity [74]. Powell et al. [76] further demonstrated that post-weaning *Gpr75* knockout mice developed leaner bodies with reduced fat deposits and increased appetite, achieving a lean phenotype without compromising overall health. Adult *Gpr75* knockout mice also exhibited improved glucose tolerance, suggesting GPR75 inhibition could benefit metabolic health by reducing energy intake and fat storage while enhancing insulin sensitivity. Hossain et al. [77] found that, under HFD conditions, *Gpr75* knockout mice gained significantly less weight, maintained lower energy expenditure compared to wild-type mice, and exhibited normal insulin signaling. Recently, Zhong et al. [78] conducted a comprehensive genetic study on the South China carp (*Cyprinus carpio rubrofuscus*) using whole-genome resequencing and RNA-seq analysis. They identified 12 candidate single nucleotide polymorphisms (SNPs), 10 of which are associated with the *Gpr75* gene. Located primarily in the promoter, intron, or untranslated region (UTR) of the gene, these SNPs suggest that genetic variations might modulate individual growth rates by influencing *Gpr75* expression levels. Furthermore, carp with reduced growth exhibited decreased *Gpr75* expression and lower insulin levels, along with elevated blood glucose, suggesting that GPR75 regulates body weight by modulating insulin metabolism.

Contrary to previous findings, Jiang et al. [42] recently observed in a *Gpr75* knockout mouse model that the lean phenotype under HFD conditions primarily results from reduced food intake regulated by neural mechanisms rather from insulin resistance. They found that although *Gpr75*^−/−^ mice displayed reduced body fat under both HFD and normal diet conditions, there were no significant abnormalities in glucose or insulin metabolism under HFD. Pair-feeding experiments confirmed that reduced food intake was the main contributor to the lean phenotype of *Gpr75*^−/−^ mice. Further analyses revealed that GPR75 is expressed across various brain regions, particularly in the primary cilia of hypothalamic cells. Given that neuronal primary cilia play a significant role in regulating energy metabolism and that mutations in cilia-associated genes have been linked to obesity [79], the study suggests that GPR75, as a ciliary-associated protein, may modulate energy expenditure and food intake through neuronal mechanisms. Additionally, Leeson et al. [80] provided further evidence for GPR75’s involvement in the neural regulation of body weight and energy balance. Using single-cell RNA sequencing and histochemical analysis, they found that GPR75 is co-expressed with key neurons in the hypothalamus, such as those producing neuropeptide Y, critical for appetite and energy regulation. Under HFD conditions, *Gpr75* knockout mice exhibited lower food intake, increased physical activity, lower liver weight, and decreased hepatic triglyceride and lipid levels, suggesting that *Gpr75* deletion protects against non-alcoholic fatty liver disease (NAFLD).

### 4.4. The Role of GPR75 in Tumor Development and Progression

**Figure 3:**
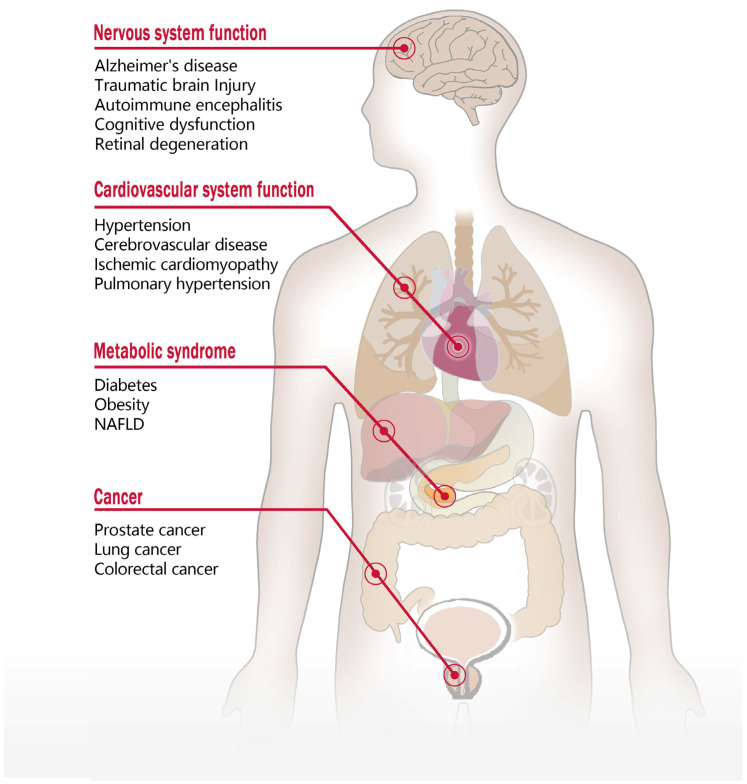
The relationship between GPR75 and related diseases. Nervous System: GPR75 provides neuroprotection in conditions such as Alzheimer’s disease, traumatic brain injury, and autoimmune encephalomyelitis, while also being essential for the function of retinal cone photoreceptor cells. *Gpr75* knockout mice exhibit increased anxiety-like behavior and memory deficits in trace fear conditioning tasks. Cardiovascular System: The 20-HETE/GPR75 axis is linked to hypertension and ischemic cardiomyopathy, with GPR75 modulation improving blood pressure and vascular function. CCL5/GPR75 pairing is associated with pulmonary arterial hypertension. Metabolic System: GPR75 regulates insulin secretion and glucose homeostasis, with knockout mice demonstrating a lean phenotype, enhanced metabolic profiles, and resistance to non-alcoholic fatty liver disease. Cancer: GPR75 contributes to the progression of prostate, lung, and colorectal cancers. Its interaction with 20-HETE may influence tumor evasion and invasion, while its methylation status could serve as a prognostic marker for lung squamous cell carcinoma.

Beyond its role in promoting vasoconstriction, regulating blood pressure, and modulating nervous and metabolic functions, 20-HETE has been shown to significantly impact tumor biology by influencing cell proliferation, migration, and invasion, which are closely associated with various cancers [81]. In the tumor microenvironment, 20-HETE modulates immune responses; Chen et al. [39] demonstrated that 20-HETE activates tumor-associated fibroblasts through the GPR75/STAT3/c-Jun signaling pathway, thereby promoting immune evasion and enhancing the invasiveness of non-small-cell lung cancer (NSCLC) cells. Hardwick et al. [73] recently reported elevated GPR75 expression in hepatocellular carcinoma (HCC) samples, despite decreased 20-HETE levels, suggesting that inflammatory responses and the tumor microenvironment in HCC may upregulate GPR75 expression via alternative pathways. Additionally, Cárdenas et al. [37] found that 20-HETE enhances metastatic properties in human prostate cancer cells by activating GPR75. This activation increases phosphorylation of key signaling molecules, including EGFR, NF-κB, AKT, and p38, and upregulates HIC-5 expression, all of which are closely linked to malignant transformation in tumor cells. Further research has established a strong correlation between GPR75 and androgen receptor (AR) expression in prostate cancer (PCa) samples [82]. Stimulation of the 20-HETE/GPR75 axis upregulates AR expression and transcriptional activity, whereas these effects on the PI3K/AKT and PKC signaling pathways can be inhibited by the 20-HETE analog 19-HEDE. This suggests that targeting the 20-HETE/GPR75 axis may provide a strategy to modulate AR-related signaling in PCa. In lung squamous cell carcinoma (SCC), Li et al. [83] found that *GPR75* gene methylation is significantly elevated, with high methylation levels correlating with shorter survival times. This suggests that the methylation status of GPR75 could serve as a potential prognostic biomarker for lung SCC, aiding in predicting patient survival outcomes. In colorectal cancer, Ghorbanzadeh et al. [84] developed a novel PEGylated liposomal delivery system for the efficient delivery of metformin. In the human colorectal cancer cell line Caco-2, cells treated with metformin-loaded liposomes exhibited significantly reduced mRNA expression levels of GPR75, Cyclin D1, Bcl-2, and hTERT compared to those treated with free metformin, while the expression of the BAX gene was upregulated. These findings suggest that metformin-loaded liposomes may inhibit cancer cell growth and proliferation by regulating the expression of genes including GPR75, thereby enhancing its antitumor efficacy [84]. The relationship between GPR75 and various diseases is shown in Figure 3.

## 5. Current Issues and Perspectives

In recent years, despite significant advancements in the deorphanization research of GPR75 and its impact on related diseases, many questions remain unanswered. First, there is ongoing debate about identifying GPR75’s natural endogenous ligands, and it is currently classified as an orphan receptor by IUPHAR [4]. Based on the existing studies, 20-HETE exhibits a higher affinity for GPR75 compared to CCL5. Pascale et al. [41] suggested that CCL5 might act as a negative regulator by potentially interfering with 20-HETE binding to GPR75. However, whether there are similar interactions among GPR75, 20-HETE, and CCL5 occur in other tissues and organs requires further investigation. Additionally, the interaction mechanism between 20-HETE and GPR75 needs validation in more tissues and organs. For example, although 20-HETE plays significant roles in both physiological and pathological processes within the heart, the precise contribution of GPR75 to 20-HETE-induced myocardial injury remains poorly defined, particularly the molecular mechanism underlying 20-HETE-mediated GPR75 activation. Moreover, it is uncertain whether GPR75 is the sole receptor for 20-HETE, given that studies have reported that 20-HETE can also activate the free fatty acid receptor 1 (FFAR1/GPR40) to promote insulin secretion [85]. Evidence suggests the possibility of different GPR75 isoforms across tissues, or even the existence of additional receptors capable of binding 20-HETE. For instance, recent studies by Yu et al. [86] have shown that 20-HETE activates platelets via Gα_q_-coupled GPCRs. However, no GPR75 expression was detected in human platelets, suggesting that 20-HETE may act through other GPCRs rather than via a GPR75-dependent pathway. Additionally, although Pascale et al. [41] demonstrated via surface plasmon resonance that 20-HETE exhibits high affinity for GPR75 and observed 20-HETE-induced β-arrestin recruitment in HTLA cells, Jiang et al. [42] could not confirm this effect in HEK293 cells. All these findings suggest that the identification of 20-HETE as an endogenous ligand for GPR75 remains highly controversial, with its mechanisms of action potentially varying across cell types or tissues. Further research is needed to elucidate GPR75’s endogenous ligands, their binding mechanisms, and the functional implications of these interactions in various pathological conditions.

Based on a systematic review of the literature, current studies on GPR75 have primarily focused on its roles in biological processes and molecular functions, whereas its dynamic subcellular localization and conformational regulatory mechanisms remain poorly understood. Although GPCRs are predominantly membrane-bound, recent studies have revealed that GPCR oligomerization and internalization induce conformational changes and dynamic intracellular relocalization, which critically influences signal transduction and the activation of biased signaling pathways [22]. Under the action of different ligands, GPCRs form oligomeric complexes with themselves or other GPCRs, allosterically alter transmembrane domain conformations, and thereby regulate the balance between G-protein-dependent and β-arrestin-mediated signaling pathways [87]. For example, dimerization of the platelet-activating factor receptor (PAFR) has been shown to enhance G protein signaling while reducing β-arrestin recruitment and agonist-induced receptor internalization [88]. Similarly, heterodimerization of D1 and D2 dopamine receptors alters ligand selectivity and signaling outcomes, offering new therapeutic opportunities for neurological disorders, such as post-traumatic stress disorder (PTSD) and Parkinson’s disease [89]. Likewise, the oligomerization of the glucagon-like peptide-1 receptor (GLP-1R) with the glucose-dependent insulinotropic polypeptide receptor (GIPR) in regulating post-endocytic insulin secretion offers a promising new approach for diabetes treatment [90]. However, as a novel GPCR, the oligomerization and internalization regulatory mechanisms of GPR75 remain poorly understood. In-depth exploration of GPR75’s oligomerization dynamics and intracellular localization will not only reveal novel mechanisms by which this receptor regulates cardiovascular homeostasis, energy metabolism, and synaptic plasticity but also establish a theoretical foundation for developing biased drugs targeting GPR75, which is of significant value in treating major diseases, such as obesity, Alzheimer’s disease, and cancer.

Although many scientific questions remain regarding the deorphanization and biological functions of GPR75, particularly concerning its activatable ligands and the factors that stimulate its downstream signaling pathways, this receptor has already demonstrated significant roles in hypertension, cardiovascular diseases, neurological disorders, and obesity-related metabolic syndrome (Table 1). Currently, no drugs targeting GPR75 have entered clinical trials, but various research efforts are underway, including the development of small molecule compounds, antibodies, and gene-silencing technologies. Recently, AstraZeneca and Regeneron initiated a collaboration to develop GPR75-targeting gene-silencing drugs, monoclonal antibodies, and small-molecule modulators aimed at preventing obesity and its related complications. Additionally, BeBetter Med’s oral small-molecule GPR75 inhibitor, BEBT-809, is currently undergoing an investigational new drug (IND) regulatory trial, and Alnylam Pharmaceuticals is investigating siRNA drugs targeting GPR75 mRNA for the prevention and treatment of obesity and other weight-related diseases [91]. Recently, researchers from Shuimubiosciences [92] used cryo-electron microscopy to resolve the high-resolution 3.6 Å structure of human GPR75 in complex with the nanobody NbH3, which mimics a G protein. This structure showed that NbH3 stabilizes the receptor in a quasi-active state, revealing notable conformational changes in the transmembrane domains. The orthosteric binding pocket, composed of both polar and hydrophobic residues, provides critical structural insights for guiding future drug design. In conclusion, as a typical GPCR, GPR75 holds great promise as a drug target. The development of GPR75 agonists or antagonists may lead to major advances in the treatment of obesity, cancer, Alzheimer’s disease, cardiovascular disorders, and other conditions.

## Figures and Tables

**Table 1:** The deorphanization research progress of GPR75.

Year	Reference	Putative Ligands	Related Disease	Major Findings
1999	Tarttelin [8]	-	-	The *GPR75* gene was first identified in the human genome.
2001	Sauer [10]	-	-	*GPR75*, primarily expressed in the CNS and retina, shows no link to age-related macular degeneration.
2006	Ignatov [5]	CCL5	Alzheimer’s disease	CCL5, an endogenous ligand for GPR75, activates the MAPK/AKT pathway to inhibit Aβ-induced neuronal apoptosis.
2008	Lin [64]	-	Glaucoma	The 2p16 region containing *GPR75* is linked to POAG, though GPR75 may not be a direct cause.
2013	Liu [17]	CCL5	Diabetes	GPR75 is expressed in pancreatic β cells, where CCL5 enhances insulin secretion and glucose homeostasis by this receptor.
2013	Southern [25]	-	-	CCL5/GPR75 pairing was not confirmed by β-arrestin recruitment assays.
2014	Kakarala [9]	-	-	GPR75 shares transmembrane domain homology with several receptors across species, including neuropeptide Y and galanin receptors.
2017	Garcia [6]	20-HETE	Hypertension	*Gpr75* is expressed in the cardiovascular system, where 20-HETE activates it to induce endothelial dysfunction and VSMC contraction, contributing to hypertension.
2018	Dedoni [12]	CCL5	-	CCL5 activates GPR75 via internalization, while *Gpr75* knockdown blocks CCL5-induced pERK activation in SH-SY5Y cells.
2018	Sedláková [66]	20-HETE	Hypertension	AAA, a 20-HETE receptor antagonist, reverses malignant hypertension by blocking GPR75 in transgenic rats.
2018	Voogdt [11]	-	-	In chickens, *Gpr75* is adjacent to *Tlr15*, suggesting similar biological functions.
2019	Gençoğlu [18]	CCL5	Diabetes	CCL5 promotes insulin secretion in MIN6 cells through GPR75.
2019	Li [83]	-	Lung cancer	In lung SCC, *GPR75* hypermethylation serves as a prognostic marker.
2020	Cárdenas [37]	20-HETE	Prostate cancer	20-HETE activates EGFR/AKT signaling via GPR75, enhancing prostate cancer metastasis.
2020	Gonzalez-Fernandez [47]	20-HETE	Cerebrovascular disease	*Cyp4a* and *Gpr75* are co-expressed in the brain, where 20-HETE regulates VSMCs and pericytes via GPR75.
2020	Mao [46]	20-HETE	Ischemic cardiomyopathy	In H9c2 cardiomyocytes, GPR75 mediates 20-HETE-induced apoptosis through mitochondrial damage.
2020	Gawrys [67]	20-HETE	Hypertension	AAA and EET-A improve endothelial function and lower blood pressure in hypertensive rats.
2021	Gilani [38]	20-HETE	Diabetes	In 3T3-L1 adipocytes, 20-HETE activates GPR75 to inhibit insulin receptor phosphorylation, leading to insulin resistance.
2021	Pascale [41]	20-HETE	-	20-HETE is a high-affinity ligand, while CCL5 is a low-affinity negative regulator of GPR75.
2021	Akbari [74]	-	Obesity	GPR75 truncating variants are linked to BMI, and its knockout in mice prevents weight gain and improves glycemic control on a high-fat diet.
2022	Agostinucci [43]	20-HETE	Hypertension	AAA reduces 20-HETE-dependent hypertension in *Cyp4a12* transgenic mice.
2022	Tunctan [44]	20-HETE	Hypotension and tachycardia	The 20-HETE mimetic 5,14-HEDGE protects against LPS-induced hypotension via GPR75, reversed by AAA.
2022	Chen [39]	20-HETE	Lung cancer	20-HETE upregulates GPR75 and activates fibroblasts, promoting immune evasion and invasiveness in lung cancer.
2022	Powell [76]	-	Obesity	*Gpr75* knockout mice show reduced weight and improved glucose tolerance and insulin sensitivity.
2022	Liu [45]	20-HETE	Ischemic cardiomyopathy	GPR75 mediates 20-HETE-induced cardiomyocyte apoptosis via Ca^2+^ overload and ROS overproduction in NRCMs.
2022	Lv [92]	-	-	Cryo-EM revealed the 3.6 Å structure of GPR75, detailing its ligand-binding pocket for drug design.
2022	Pascale [40]	20-HETE	-	20-HETE and agonists activate GPR75, while 20-SOLA, AAA, and antagonists inhibit its activity.
2023	Hossain [77]	-	Obesity	*Gpr75*-deficient mice resist weight gain on a high-fat diet without affecting insulin signaling.
2023	Speidell [63]	-	Cognitive dysfunction	*Gpr75* knockout mice show anxiety and memory deficits linked to hippocampal protein dysregulation.
2023	Vasudevan [65]	-	Retinal degeneration	*Gpr75* knockout causes age-related cone photoreceptor loss and dysfunction.
2023	D’Addario [19]	CCL5	Pulmonary hypertension	In hypoxia-induced PH, CCL5 causes IPA contraction by decreasing cAMP levels via the GPR75/Gα_i_ pathway.
2023	Cárdenas [82]	20-HETE	Prostate cancer	In prostate cancer, *Gpr75* expression correlates with AR, and 20-HETE/GPR75 axis increases AR activity.
2023	Ghorbanzadeh [84]	-	Colorectal cancer	Metformin reduces *Gpr75* expression, potentially suppressing colon cancer growth.
2024	Leeson [80]	-	NAFLD	*Gpr75* co-expressed in hypothalamic neurons regulates appetite and contributes to fatty liver disease.
2024	Jiang [42]	-	Obesity	In hypothalamic cilia, *Gpr75* regulates food intake and energy, with its loss promoting a lean phenotype.
2024	Ma [59]	20-HETE	Traumatic brain injury	The 20-HETE/GPR75 axis drives TBI-induced inflammation, reversed by *Gpr75* knockdown or HET0016.
2024	Zhong [78]	-	Obesity	In growth-retarded carp, reduced *Gpr75* expression impairs insulin metabolism, affecting body weight.
2024	Hardwick [73]	20-HETE	MASLD	The 20-HETE/GPR75 axis is involved in regulating the progression of MASLD.
2025	Han [36]	20-HETE	Myocardial hypertrophy	The 20-HETE/GPR75 axis is involved in Ang II-induced cardiomyocyte hypertrophy.

Abbreviations: GPR75: G-protein-coupled receptor 75; *Gpr75/GPR75*: the gene name of GPR75; CNS: central nervous system; CCL5: C-C motif chemokine ligand 5; AKT: protein kinase B; Aβ: amyloid β-protein; POAG: primary open-angle glaucoma; 20-HETE: 20-hydroxyeicosatetraenoic acid; VSMCs: vascular smooth muscle cells; AAA: N-disodium succinate-20-hydroxyeicosa 6(Z),15(Z)-diencarboxamide; EGFR: epidermal growth factor receptor; EET-A: epoxyeicosatrienoic acids; SCC: squamous cell carcinoma; BMI: body mass index; 5,14-HEDGE: N-(20-hydroxyeicosa-5[Z],14[Z]-dienoyl) glycine; LPS: lipopolysaccharide; NRCMs: neonatal rat cardiomyocytes; ROS: reactive oxygen species; CYP4A: cytochrome P450 4A; *Cyp4a/Cyp4a12*: the gene name of CYP4A; PH: pulmonary hypertension; IPAs: intrapulmonary arteries; AR: androgen receptor; NAFLD: non-alcoholic fatty liver disease; TBI: traumatic brain injury; MASLD: metabolic dysfunction-associated steatosis liver disease.
